# Race-based trauma and post-traumatic growth through identity transformation

**DOI:** 10.3389/fpsyg.2023.1031602

**Published:** 2023-02-08

**Authors:** Dorothy Chin, Amber M. Smith-Clapham, Gail E. Wyatt

**Affiliations:** Department of Psychiatry and Biobehavioral Sciences, Semel Institute for Neuroscience and Human Behavior, University of California, Los Angeles, Los Angeles, CA, United States

**Keywords:** race-based trauma, post-traumatic growth, identity narratives, Black Americans, Asian Americans

## Abstract

Race-based trauma has been linked to multiple adverse health and mental health outcomes, including hypertension, post-traumatic stress, anxiety, and depression. While the possibility of post-traumatic growth (PTG) has been investigated following other types of trauma, relatively less work has been done on PTG following race-based trauma. In this article, we present a theoretical framework integrating three areas of research: race-based trauma, PTG, and racial identity narratives. Based on the work on Black and Asian American identity and integrating theory and research on historical trauma and PTG, this framework posits that the transformation of externally imposed narratives into more authentic, internally generated ones can serve as an important influence that sparks PTG after racial trauma. Based on this framework, strategies and tools that enact the cognitive processes of PTG, including writing and storytelling, are suggested as ways to promote post-trauma growth in response to racial trauma.

## Introduction

Race-based trauma is increasingly recognized within psychology as a distinct type of trauma, resulting in post-traumatic stress symptoms (Chin et al., 2020, p. 9), including hyper-vigilance (Carter et al., 2005; Carter and Forsyth, 2010) and dissociation (Polanco-Roman et al., 2016), as well as depression, generalized anxiety, and somatic symptoms (Noh et al., 1999; Belle and Doucet, 2003; Williams et al., 2003, 2018; Alegría et al., 2008). The evidence for adverse health and mental health effects owing to racial discrimination is overwhelming, consistently found among diverse samples of African Americans, Latin Americans, and Asian Americans (Gee et al., 2007, p. 2; Lee and Ahn, 2013; Nadimpalli et al., 2014; Torres and Vallejo, 2015). In addition, race-based trauma, unlike other types of trauma, has the distinction of being a common occurrence in the lives of targeted individuals, yet with potentially damaging consequences (Carter et al., 2016; Polanco-Roman et al., 2016). The accumulation of racial stress predisposes individuals to psychological symptoms and disorders (Gee et al., 2007; Williams et al., 2021). Moreover, racial microaggressions, a subtle and covert form of prejudice or discrimination, have been linked to depression symptoms and negative affect (Torres et al., 2011; Nadal et al., 2014).

With recent geopolitical events instigating a rise in race-based violence in the United States, there is an urgent need for greater attention to racial trauma on the part of researchers and mental health professionals. While hate crimes against African American citizens span the history of the United States, the rise in the open expression of white supremacist ideology has precipitated recent mass shootings targeting African Americans in Buffalo, Milwaukee, and other U.S. cities (Collins, 2020; Rahman, 2021). The brutal killings of George Floyd, Breonna Taylor, and other Black citizens have triggered mass outcry and protests worldwide (McLaughlin, 2020). Additionally, attacks on Asian Americans rose 149% in 2020, sparked by racial slurs used by political leaders blaming Asians for COVID-19 [Center for the Study of Hate and Extremism (CSUB), 2020; Gover et al., 2020, pp. 649, 653–663]. In addition to the direct damage to the targeted individual’s psychological health, racial violence appears to have spillover effects onto the surrounding community. For example, a recent study found that when an unarmed Black citizen is killed by the police, the surrounding population evidenced poorer mental health in the 2 months following the incident, compared to incidents in which an armed Black citizen is killed or an unarmed White citizen is killed (Bor et al., 2018). These spillover effects of racial trauma are also exemplified by the widespread distress evidenced in the Asian American community during the COVID-19 pandemic. As hate crimes on Asian Americans have risen, mental and emotional distress among Asian Americans has increased accordingly (Cheah et al., 2020; Hyun et al., 2021), including among those who did not personally experience an attack (Wu et al., 2020). Furthermore, constant vigilance evoked by race-related threat has been associated with cumulative stress and depression symptoms across diverse minorities groups (Woody et al., 2022). In a survey conducted in 2022, nearly one-third of Black Americans (32%) and one-fifth (21%) of Asian Americans said they worried every day or almost every day that they might be threatened or attacked because of their race or ethnicity, compared to 4% of White Americans, and many more changed their everyday routines to avoid possible attacks (Pew Research Center, 2022). Consistent with the effects of exposure to racism in the context of a society in which racism pervasive, the Diagnostic and Statistical Manual-V (DSM-V) has expanded its diagnostic criteria for Post Traumatic Stress Disorder (PTSD) to include instances in which a person, “witnesses a traumatic event occurring to someone else or learns of a traumatic event that occurred to a close other” (American Psychiatric Association, 2013, p. 271).

Post-traumatic growth (PTG) is a construct that attempts to capture the positive psychological change that may ensue after a traumatic experience (Calhoun and Tedeschi, 1999). While resilience refers to an individual’s ability to cope with trauma and return to the pre-trauma state of functioning (Smith et al., 2019, p. 3), PTG describes a profound change within an individual in which a traumatic experience prompts deep questioning, deconstruction, and reconstruction of one’s basic schemas about oneself and about life, ultimately resulting in personal growth (Calhoun and Tedeschi, 2004, pp. 99–101). Some manifestations of growth include more meaningful relationships, a greater appreciation for life, an increased sense of personal strength, deeper spirituality, and a recognition of new directions and goals for one’s life (Calhoun and Tedeschi, 2004, p. 95; Smith et al., 2019, p. 4). Studies have shown that promoting PTG may be more important for supporting wellbeing after trauma than reducing post-traumatic symptoms (Hamby et al., 2022).

According to the Affective-Cognitive Processing Model of PTG (Joseph et al., 2012), the process that underlies PTG involves repeated appraisals of the traumatic event, with each appraisal instantiating challenges to one’s previously existing schemas, which prompts resolutions to the fractured schema until it is reconstructed in a new, meaningful manner. Growth occurs when the individual is able to reflect on the trauma from a relative distance and create meaning from the experience. Empirical research based on this model further elucidates this process, in that two distinct types of rumination in the appraisal process emerged: intrusive rumination, which occurs with volition, and deliberative rumination, which refers to intentional reflection (Tedeschi and Blevins, 2015). Studies have shown that both types may be necessary for PTG, in that intrusive rumination soon after the traumatic experience predicts deliberative rumination (Cann et al., 2011), and that both higher levels of both may predict PTG (Calhoun et al., 2000; Tedeschi and Blevins, 2015).

This model of PTG is also consistent with studies demonstrating that higher levels of trauma may be needed to achieve growth (Jirek and Saunders, 2018). The notion that significant levels of trauma is necessary for PTG is supported by studies showing that higher PTSD symptoms predicted higher PTG (Hyun et al., 2021). However, levels that are too extreme may be inhibiting (Kleim and Ehlers, 2009; Kunst, 2010). That is, when the event is not significant enough for individuals to question and deconstruct their existing schemas, no growth happens. However, if trauma is so extreme as to overwhelm individuals’ cognitive capacity to reflect deliberately, they are likely to be “stuck” in intrusive rumination and unable to progress to deliberative rumination and meaning-making (Joseph et al., 2012). Thus, the ideal circumstance for PTG to occur is when the event is significantly traumatic to trigger reflection, yet leaving the individual with enough coping ability and resources to make meaning of the event.

## Race-based trauma and post-traumatic growth

While PTG is a reliably demonstrated phenomenon, its relationship to race-based trauma remains less so. Indeed, the very notion of “post” traumatic growth poses a challenge with respect to racial trauma, as such events are likely to be persistent, recurrent, and cumulative rather than one-time, discrete events. The fact that traumagenic events are now understood to encompass observed or vicariously experienced events (American Psychiatric Association, 2013) captures the ubiquitous and open-ended nature of race-based trauma for racial minorities. The demonstrated spillover effects onto the community when an unarmed Black citizen dies at the hands of the police (Bor et al., 2018) and the long list of people who have died that way (Rahman, 2021) support this viewpoint. How, then, can PTG be reconciled with race-based trauma, when it is not “post,” and trauma is collective, pervasive, and persistent? Theories on historical trauma, focusing on mass-level collective, multigenerational, and transgenerational trauma such as the colonization and eradication of Indigenous peoples, provide frameworks that have significant overlap with racial trauma (Bryant-Davis and Ocampo, 2005; DeGruy-Leary and Robinson, 2005; Hyatt-Burkhart and Lopez Levers, 2012; Mohatt et al., 2014; Williams-Washington and Mills, 2018; Ortega-Williams et al., 2019). To illustrate, the Spokane/Coeur D’Alene writer Sherman Alexie alludes to the unmitigated transgenerational grief and the erasure of cultural identity that stems from the disappearance of the wild salmon, therein linking historical loss with contemporary suffering:

“After the Grand Coulee Dam murdered our wild salmon, we stopped being Spokane Indians and became a Paraphrase of Spokane Indians/Our identity has been clarified for us/We are the Unsalmon People/We are Unsalmon/We are Un” (Alexie, 2017, p. 160).

The entrenched grief that spans centuries among subjugated and colonized peoples requires a distinct strategy to promote PTG. Ortega-Williams et al. (2021) postulate several important elements in this process: (a) deliberative rumination; (b) creating redemptive narratives; (c) discovering deeper meaning. As noted earlier, this has much overlap with models of PTG that do not explicitly address historical or racial trauma, particularly in the elements of deliberative rumination and meaning-making. Where they differ may be an emphasis on creating redemptive narratives, that by using storytelling and action, oppressed groups and individuals may reclaim cultural tools, values, and priorities (Ortega-Williams et al., 2021). While this framework applies to contemporary as well as historical trauma, according to the authors, it has been criticized for over-emphasizing the legacy of past violence and insufficiently addressing ongoing exposure to current structural violence (Kirmayer et al., 2014).

## Racial identity

Racial/ethnic identity is an aspect of one’s self concept derived from a sense of belonging and commitment to a particular racial or ethnic group, and includes self-identification, pride and positive valuation of the group, and taking part in the group’s traditions, practices, and values (Phinney, 1990; Ashmore et al., 2004). It has been posited as a protective factor in mental health (Phinney, 1990; Cokley, 2007), associated with fewer depressive symptoms (Tummala-Narra, 2015), less stress (Espinosa et al., 2018), and higher self-esteem (Phinney et al., 2001; Umaña-Taylor et al., 2004), perhaps because individuals would attribute discrimination directed at them not to their personal characteristics but to social injustice (Lee and Ahn, 2013). However, a strong racial identity might also lead one to perceive more discriminatory behavior and to interpret ambiguous incidents as discriminatory (Sellers and Shelton, 2003), as has been found in a meta-analysis which concluded that for Black Americans, a greater sense of racial belonging was associated with more perceived discrimination, indirectly affecting psychological distress (Lee and Ahn, 2013). A recent large-scale study found that high racial identification intensified the negative mental health effects of racial discrimination for American Indians/Alaska Natives, and Latinx individuals, while moderate racial identity served as a buffer for Asian Americans and Black Americans (Woo et al., 2019). Furthermore, other studies have found that those with great racial salience are more able to identify racism when it occurs, which triggers the stress response (Franklin-Jackson and Carter, 2007). Thus, racial identity may be a mediator or moderator through which race-based trauma leads to eventual outcomes, determining whether deterioration, little or no effect, or growth occurs in the aftermath of trauma. As such, racial identity narratives may serve as a lever upon which PTG may be nurtured and developed. In the following section, we explore the concept of identity narratives and describe the narratives externally imposed upon Asian Americans and Black Americans throughout American history, based on the Racial Triangulation Theory of Kim (1999) and their relationship to racial trauma. We then explain how transforming these externally imposed identity narratives into internally generated, more authentic identities can catalyze healing and create PTG.

## Identity narratives

Personal narratives are stories constructed by individuals about their lives that give meaning and define who they are (McAdams et al., 2006; McLean, 2008). The resulting identity, termed narrative identity, is the “internalized and evolving story of the self” (McAdams, 2011). There exists a vast literature examining the multitude of issues related to the development and importance of narrative identity; particularly relevant to our purposes here is one involving the authorship of stories, namely, individual vs. society authorship. According to McAdams (2018), narrative identity is constructed through an iterative process of experiencing events, narrating these experiences to others, editing their narratives in response to others’ reactions, experiencing new events, and so on. Rarely, if at all, is the authorial process done with complete freedom but rather within a historical and cultural context, with its specific constraints and opportunities (McAdams, 2018). Herein lies the influence of externally imposed narrative identities, especially salient for minoritized individuals, who experience and may internalize the constant exposure to racist narratives about their group over time. Indeed, individuals draw heavily on the “prevailing cultural norms and the images, metaphors, and themes” they encounter in their social contexts (McAdams, 2011).

The racial themes encountered by minoritized groups in America throughout its history have been extensively discussed elsewhere and their full accounting is beyond the scope of this analysis. We endeavor to focus on two examples—Black Americans and Asian Americans—and to highlight the aspect most relevant to our thesis, namely, the externally imposed identity narratives and their role in exacerbating racial trauma or enhancing PTG^1^. Dating back to the early 1600’s when the first Black Americans were abducted from Africa and brought to the United States to work as slaves, the myth that Black Americans were inferior relative to Whites has been propagated and perpetuated. This “less than” narrative was, at its most literal, codified in the United States Constitution, wherein Black individuals were considered to be three-fifths of a White individual, and continuing to the present day, encompassing virtually every domain of human ability and enterprise. Much has been written about the blatant stereotyping of Black men as more violent and criminal (Oliver, 2003). More recent identity narratives imposed upon Black Americans include being less intelligent, less articulate, lower-achieving, and possessing lesser leadership ability than other ethnoracial groups (Sue et al., 2007; Herrnstein and Murray, 2010; Robinson-Wood et al., 2015; Taylor et al., 2019).

The history of Asian Americans in the United States dates back to the 1800’s, when large numbers were brought in primarily from China as exploited labor to work on the transcontinental railroad and in the mines, and from Japan and the Philippines to work on sugar plantations in Hawaii (Okihiro, 2005). Subjected to lynchings and other forms of racial violence and discrimination, these groups were nevertheless instrumental in the building of American Infrastructure and Society (Choy, 2022). However, in spite of Asian Americans’ integral contributions and long histories in this country, the identity imposed upon them to the present day remains one of a foreigner. For example, “where are you from?” Is a question that many Asian Americans, to this day, have been asked more than once, along with, “do you speak English?” (Wu, 2002; Sue et al., 2007). While Black Americans have had to contend with the externally imposed identity narrative of inferiority, Asian Americans have been long viewed as the perpetual foreigner. Research has shown that these identity narratives, when internalized, harm the psychological wellbeing of individuals. For example, when Black youth perceive lower regard from the public, the discrimination they experienced on the previous day were associated with an increase in depressive symptoms (Seaton and Iida, 2019). This finding is consistent with the belief that the broader society views Black Americans in a negative light (Sellers et al., 2006) as well as the internalization of these views (Cross et al., 2017). On a cultural level, these narratives perpetuate racism and race-based violence and discrimination, as they justify racist behavior and inequitable social conditions.

**FIGURE 1:**
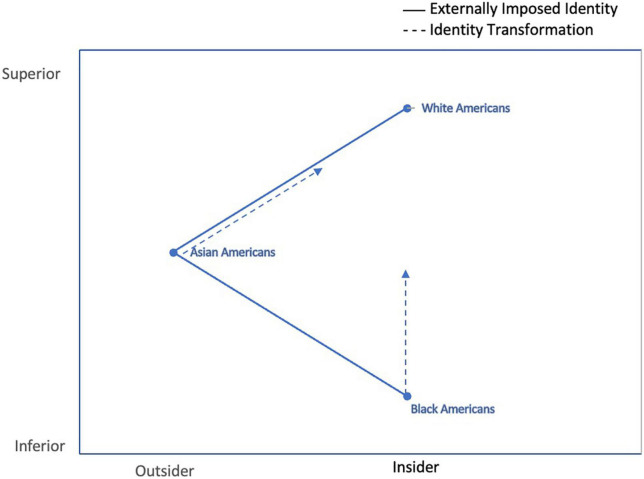
Adapted from Kim (1999) Racial Triangulation Theory.

Based on the histories of Black and Asian Americans in the United States, Kim’s (1999) Racial Triangulation Theory describes the relative positions of Black Americans and Asian Americans to Whites according to two dimensions: superior/inferior and foreigner/insider (see Figure 1). Along these two axes, Black Americans are positioned at the inferior and insider end, Asian Americans are placed in extreme foreigner area and the middle of the superior/inferior axis, while White Americans are high on both superiority and insider status. The positioning of these groups keep both groups “in check,” beneath the status of White Americans while pitting one against the other, thus accruing economic and other advantages to White Americans (Kim, 1999). While Kim’s theory focuses on Black and Asian Americans, the theory may be reasonably applied to other minoritized groups as well. For example, Latin American groups may be viewed in the middle of both axes, less foreign than Asian Americans and less inferior than Black Americans, though lesser than White American, and Indigenous peoples would plausibly be insiders but inferior to White Americans. While the sociological analysis is explicated elsewhere (e.g., Kim, 1999), the psychological implications are clearly relevant to our proposed framework and supported by empirical research: these externally imposed identities cause abiding psychological harm, in the form of post-traumatic stress, depression, and other disorders. In the face of racial trauma, these identities are triggered and likely to cause further psychological damage. Within our framework, we postulate that the transformation of externally imposed identity narratives into internally generated, integrated, and culturally aligned ones has tremendous potential to heal racial trauma and spark PTG.

## Transforming racial identity narratives to foster post-traumatic growth

**FIGURE 2:**
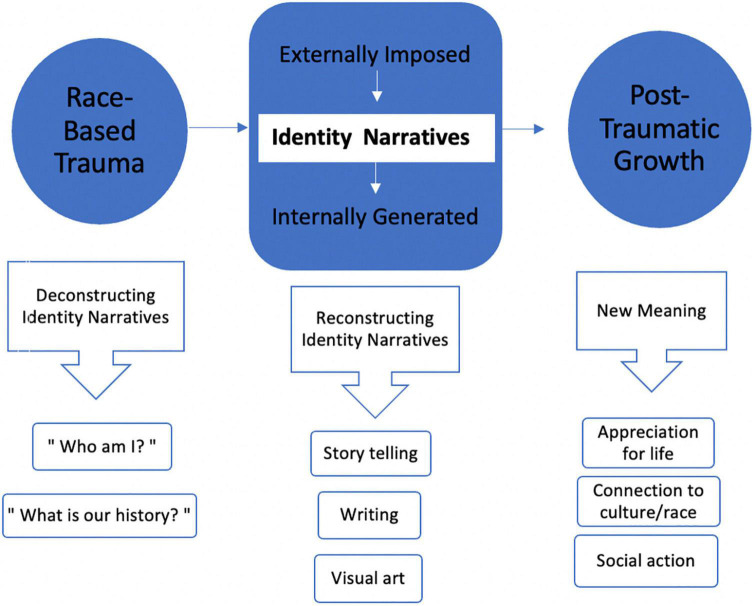
Framework for post-traumatic growth following race-based trauma.

As shown in Figure 2, identity transformation is the mechanism through which race-based trauma can lead to PTG. Consistent with the Affective-Cognitive Processing Model of PTG (Joseph et al., 2012), the experience of being targeted because of one’s race may trigger the questioning of one’s identity and the deconstructing of that identity. This is also consistent with the idea of an “encounter” in Cross’ model of racial identity development (Cross and Vandiver, 2001), which refers to a stimulus that triggers identity exploration, as well as research on adolescent identity development, in which identity is observed to undergo reconfigurations in accordance to shifts in contexts (Hughes et al., 2006). For example, if one has previously lacked a historical awareness of racism, a shift in the social context such as heightened attention to racial attacks, whether directly or indirectly experienced, may trigger a cascade of questions about one’s racial position (e.g., “I’m not as safe as I once thought”). In addition, the experience may lead to a new grief and an awareness of identity as located in a specific culture and history (e.g., “what is my group’s history?”). This process is fundamentally one of deconstructing of one’s narrative identity from that which is externally imposed to one that is internally generated. Vis a vis the Racial Triangulation Theory (Figure 1), for Black Americans, the inferiority narrative may be acknowledged as a basis for the racial trauma the individual has experienced and reconstructed toward greater superiority through the active countering of the narrative. For Asian Americans, the transformation process moves the outsider narrative toward the insider using various means, including bolstering knowledge of Asians in American history and engaging in projects that highlight their intertwined history with that of the country. Similarly, for Latin and Indigenous Americans, the rejection of outsider and inferior statuses and the building of an identity congruent with their true cultural history may be a pivotal step toward PTG.

The internalization of inferiority beliefs has been hypothesized as the mechanism by which racial discrimination influences psychological functioning for Black Americans (Jones, 2000). Thus, in that context, identity is the fulcrum by which change can happen, encompassing both temporal (past and present) and social (individual and the collective) perspectives. This process addresses the issue of “post” traumatic growth being an awkward concept as applied to race-based trauma, as racism is insidious and ongoing. While externally imposed identity narratives may continue to evolve in society (e.g., the crude racial stereotypes in the past century replaced by more sophisticated racist narratives), strengthening one’s own racial identity internally continues to be an imperative in the context of pervasive racist assaults on one’s identity. The focus on racial identity narratives also aligns with the historical trauma framework (Ortega-Williams et al., 2021), which deals with mass-level, intergenerational trauma such as genocide, and which has been criticized for overlooking the effects of present-day racial trauma (Kirmayer et al., 2014), as questions about one’s racial identity will likely raise the collective history of the group.

Research on narrative identity focuses on verbal tools to construct and reconstruct narratives (e.g., Adler et al., 2017; McAdams, 2018). The notion of self-labeling and the re-appropriation of one’s identity has been linked to physical and psychological wellbeing (Wang et al., 2017; Cervone et al., 2021). As a means to create and strengthen that identity, oral storytelling, and autobiographical writing have been used as part of clinical interventions (Davis et al., 2021). Similarly, Pennebaker’s expressive writing paradigm (Pennebaker and Chung, 2011; Pennebaker and Smyth, 2016) has shown robust positive effects on health and emotional wellbeing 3 months later. These effects have been found in a plethora of studies of traumatic experiences ranging from severe traumas such as the Holocaust and sexual assault to less severe ones such as losing a job (Pennebaker and Chung, 2011). In trauma writing interventions, participants are asked to write freely without structured prompts about their trauma over a period of time. While the health effects of expressive writing have been widely demonstrated, the mechanism by which these outcomes are effected have been unclear, and multiple pathways, including cognitive, emotional, social, and biological, have been implicated (Pennebaker, 2004). One significant pathway may be the adoption of a new perspective, as words indicating insight or new understanding (e.g., “I realized”; “I see now”) were correlated with positive outcomes (Pennebaker and Chung, 2011).

In studies on the evolution of narrative identity over the life course, McAdams (2018) has used a semi-structured interview format with specific prompts that highlight key aspects of a person’s life, including: (1) high point; (2) low point; (3) turning point; (4) challenges; and (5) dreams, hopes, and plans for the future. Both formats may be integrated and adapted to reconstruct one’s racial identity narrative following race-based trauma through a series of questions or prompts; for example, “what was your first memory that included an awareness of your racial background?” A critical prompt would be one that centers around the pivotal race-based trauma, per Pennebaker’s studies, and the breakdown of assumptions that lead to a new perspective and narrative. Specifically, with respect to Black Americans and the externally imposed inferiority narrative, one prompt might ask “what are some views about Black Americans that others seem to hold?” To stimulate an internally generated narrative: “what are your views?” Similar prompts may be used for Asian Americans and other minoritized groups. Through this process, participants are doing the cognitive work of reappraising the incident, apropos the aforementioned PTG theories (Joseph et al., 2012; Tedeschi and Blevins, 2015), which also aligns with the Historical Trauma framework of deliberative rumination, creating redemptive narratives, and discovering deeper meaning (Ortega-Williams et al., 2019). From a cognitive perspective, while engaging in multiple appraisals, one’s identity narrative is deconstructed and reconstructed.

While “narrative” implies a verbal representation of a life experience and serves as the focus of this article, it is worth mentioning that identity may be constructed through non-verbal means as well, particularly for those with a greater affinity toward visual or aural representations. In the field of art therapy, it is recognized that cultural identity is communicated through art, and through visual art (Mauro, 1998). The evolution of identity may be illustrated through drawing, painting, videos, and other media (Kaimal and Arslanbek, 2020).

Once identity reconstruction and transformation begins to take root, elements of PTG are hypothesized to follow (see Figure 2), consistent with those outlined in PTG research: a new sense of meaning and purpose, increased pride and appreciation for one’s race and culture, and enhanced connection to others. These values and priorities may be enacted in one’s daily life in behaviors such as greater engagement in a community and social action.

## Discussion and future directions

This paper presents a theoretical framework by which PTG following racial trauma may be fostered through identity transformation. Many inter-relationships among these constructs have been postulated and they await empirical testing. Does transforming racial identity narratives toward internally generated ones lead to PTG? Which elements of PTG result? In regards to clinical implications, structured or semi-structured programs based on this framework may be used in settings such as college counseling centers using the tools described above. While this process may happen organically for some, others may need direction and support to move through these steps, requiring interventions that explicitly target such growth. Given that racial incidents occur regularly and psychological damage has been shown to accumulate, interventions need to be developed and sustained on an ongoing basis and not only in response to a highly publicized incident. Furthermore, various modalities of interventions need to be widely available, including formal structured programs as well as informal community “check-ins,” offered in-person and virtually. Technology may be leveraged to make resources and networks more accessible. In light of the ubiquitous nature of race-based trauma and their pernicious effects, the facilitation of PTG after such trauma should be a high priority and a vital cornerstone in efforts toward racial justice.

## Author contributions

DC conceptualized the framework, did the background research and literature review, and wrote the manuscript. AS-C constructed the figures, conducted the literature review, compiled references, and edited the manuscript. GW helped the conceptualize and reviewed the manuscript. All authors contributed to the article and approved the submitted version.

## Footnotes

1 In focusing on Black Americans and Asian Americans, we do not mean to imply that the degree of racism toward these groups are equal or their suffering comparable, nor to imply that Latinx, Indigenous Americans, and other groups have not also been targets of racism in the United States.
